# Brain Computer Interface Treatment for Motor Rehabilitation of Upper Extremity of Stroke Patients—A Feasibility Study

**DOI:** 10.3389/fnins.2020.591435

**Published:** 2020-10-21

**Authors:** Marc Sebastián-Romagosa, Woosang Cho, Rupert Ortner, Nensi Murovec, Tim Von Oertzen, Kyousuke Kamada, Brendan Z. Allison, Christoph Guger

**Affiliations:** ^1^g.tec Medical Engineering Spain SL, Barcelona, Spain; ^2^g.tec Medical Engineering GmbH, Schiedlberg, Austria; ^3^Institute of Medical Psychology and Behavioral Neurobiology, University of Tübingen, Tübingen, Germany; ^4^International Max Planck Research School for Neural & Behavioral Sciences, Tübingen, Germany; ^5^Department of Neurology 1, Kepler Universitätsklinik, Linz, Austria; ^6^Asahikawa Medical University, Hokkaido, Japan; ^7^Department of Cognitive Science, University of California, San Diego, San Diego, CA, United States

**Keywords:** brain computer interfaces, BCI, stroke, neurorehabilitation, functional electrical stimulation, upper limb, Fugl-Meyer assessment

## Abstract

**Introduction:**

Numerous recent publications have explored Brain Computer Interfaces (BCI) systems as rehabilitation tools to help subacute and chronic stroke patients recover upper extremity movement. Recent work has shown that BCI therapy can lead to better outcomes than conventional therapy. BCI combined with other techniques such as Functional Electrical Stimulation (FES) and Virtual Reality (VR) allows to the user restore the neurological function by inducing the neural plasticity through improved real-time detection of motor imagery (MI) as patients perform therapy tasks.

**Methods:**

Fifty-one stroke patients with upper extremity hemiparesis were recruited for this study. All participants performed 25 sessions with the MI BCI and assessment visits to track the functional changes before and after the therapy.

**Results:**

The results of this study demonstrated a significant increase in the motor function of the paretic arm assessed by Fugl-Meyer Assessment (FMA-UE), ΔFMA-UE = 4.68 points, *P* < 0.001, reduction of the spasticity in the wrist and fingers assessed by Modified Ashworth Scale (MAS), ΔMAS-wrist = -0.72 points (*SD* = 0.83), *P* < 0.001, ΔMAS-fingers = -0.63 points (*SD* = 0.82), *P* < 0.001. Other significant improvements in the grasp ability were detected in the healthy hand. All these functional improvements achieved during the BCI therapy persisted 6 months after the therapy ended. Results also showed that patients with Motor Imagery accuracy (MI) above 80% increase 3.16 points more in the FMA than patients below this threshold (95% CI; [1.47–6.62], *P* = 0.003). The functional improvement was not related with the stroke severity or with the stroke stage.

**Conclusion:**

The BCI treatment used here was effective in promoting long lasting functional improvements in the upper extremity in stroke survivors with severe, moderate and mild impairment. This functional improvement can be explained by improved neuroplasticity in the central nervous system.

## Introduction

Stroke is the second largest cause of death worldwide and one of the most common causes of disability (Hachinski et al., 2010). Stroke causes devastating effects in many survivors, including severe motor and sensory impairment that hinder their activities of daily living (Kim et al., 2020). The clinical consequences after a stroke vary, depending largely on the location and the specific cause of the damage (Prabhakaran et al., 2008). Stroke treatment may entail different devices and methods, but physical therapy is a central component of the rehabilitation process. The main objective of this process is to integrate the subject in the daily living activities where the subject can end up actively participating in society. Usually, the rehabilitation treatment is customized for each patient, making it impossible to find a generic protocol that is ideal for all different cases. However, each specific treatment approach must be proven by clinical evidence before it can be used in clinical routine. Constraint Induced Movement Therapy (CIMT), Neuromuscular Stimulation (NMS) or mental practice with Motor Imagery (MI) are some of the most common treatments for motor rehabilitation of the hemiplegic arm after stroke, and their efficacy has been well-established (Veerbeek et al., 2014). However, all these techniques have some important limitations, especially for patients in chronic stage with moderate or severe impairment (Winstein et al., 2016). For example, almost 50% of the chronic patients with severe functional affectation cannot improve with CIMT (Kwakkel et al., 2015).

Hence, there is a need for improved approaches to support motor rehabilitation therapy for stroke patients, especially those in the chronic stages. Some approaches to support therapy have been gaining attention, such as robotic devices (Veerbeek et al., 2017) or Virtual Reality (VR) systems (Crosbie et al., 2006; Burke et al., 2009; Bermúdez i Badia et al., 2016; Correa-Agudelo et al., 2016). While these and other approaches often consider neuroscientific principles and have fostered understanding of how the brain improves during stroke therapy, they typically do not utilize direct measures of brain activity.

Brain Computer Interfaces (BCI) use neural activity to directly control external devices with real-time feedback. Some BCI systems synchronize neural activity with feedback devices to create closed-loop multi-modal feedback aimed at bolstering Hebbian plasticity and thereby helping to restore lost motor functions (Wolpaw et al., 2000; Daly and Wolpaw, 2008). Numerous studies have shown that BCI therapy can trigger long-lasting neurological changes and improve the motor function of the upper extremity of subacute and chronic stroke patients (Ramos-Murguialday et al., 2013, 2019; Ang et al., 2014; Pichiorri et al., 2015; Cho et al., 2016; Frolov et al., 2017; Guger et al., 2017; Biasiucci et al., 2018; Cervera et al., 2018; Lyukmanov et al., 2018).

There are different methods to record brain function, but most commonly used tool in BCI systems is the electroencephalography (EEG), which has been employed to record the activity modulations associated with MI tasks. MI has been shown to activate the primary motor cortex (M1) and related motor areas and elicits Event Related Desynchronization (ERD) and Event Related Synchronization (ERS), which reflect power changes in the mu band. Several studies have shown that stroke patients can elicit ERD/ERS during MI of their paralyzed hand and during passive movement provided by robotic assistive devices (Pfurtscheller and Aranibar, 1979; Neuper et al., 2006; Aljalal et al., 2020). Furthermore, movement-related neural activity was found to be present both in the contralateral and ipsilateral side depending on movement complexity (unilateral or bilateral) and the proximity of the muscle groups to the sagittal plane of the body (shoulder or hand). Therefore, the ipsilesional M1 is also thought to play a major role in motor recovery (Cervera et al., 2018; Peng et al., 2019).

BCI systems can be combined with different types of external devices to assist the execution and learning of movements. In the approach for movement restoration, stroke survivors perform MI exercises while wearing an EEG cap. The decoded brain oscillations can be used to move a VR avatar and/or trigger another feedback mechanism to reproduce the imagined movement with the paretic limb, such as a functional electrical stimulation (FES). Hence, rewarding feedback only occurs if the patient imagines the desired movement. This feedback loop is most effective with “closed-loop” feedback, meaning that feedback is presented in real-time, ideally through informative, clear feedback that supports effective co-adaptation between the end-user and the system.

The combination of MI-based BCIs with FES or VR has shown promising results in stroke survivors. The FES combined with other therapies seems effective for upper limb motor rehabilitation after stroke (Howlett et al., 2015; Marquez-Chin and Popovic, 2020), and could be a helpful feedback mechanism with MI BCIs. For example, Biasiucci et al. (2018) studied the importance of coherent feedback. They divided stroke patients into two groups, called BCI-FES group and the sham-FES group. The patients in the BCI-FES group only got positive feedback (FES stimulation) when the patients attempted to move the paretic hand. In the sham-FES group, the FES stimulation was delivered randomly. Only the BCI-FES group showed a significant functional improvement and an increase in functional connectivity between motor areas in the affected hemisphere. Similar results were published by Ramos-Murguialday et al. (2013). VR can help the user relearn movements lost due to stroke with immersive avatars that can demonstrate movements and (with MI BCIs) perform these movements only when the patients imagine or attempt them correctly. VR feedback can be used to employ an approach based on Mirror Therapy (MT), where the subject sees the movement in the mirror. It is important that the subjects feel a sense of “body ownership” over the virtual limbs (or the limb shown in the mirror); that is, the virtual limbs feel like each subject’s real limbs (Leeb et al., 2005; Pfurtscheller et al., 2006; Nierula et al., 2019; de Castro-Cros et al., 2020).

The objective of this study is to explore the effectiveness of a specific approach toward BCI therapy to help patients with impaired upper extremity movement due to stroke. The BCI system used in this study is called recoveriX (Ortner et al., 2012; Irimia et al., 2016, 2017). This device combines MI therapy with a VR avatar and FES that provide real-time feedback based on each patient’s EEG signals. The primary measure of this study is the Fugl-Meyer Assessment for the upper extremity (FMA-UE). We also used other established functional scales to assess grasp ability, tremor, sensitivity, pain and cognitive state.

## Materials and Methods

### Participants and Study Design

The study was approved by the Ethikkommission des Landes Oberösterreich in Austria (#D-42-17) and each participant provided written informed consent before the pre-assessment. No adverse events were reported during the entire study period. The following inclusion criteria were applied to all participants: (1) able to understand written and spoken instructions, (2) stable neurological status, (3) willing to participate in the study and to understand and sign the informed consent, (4) able to attend recording sessions. The following inclusion were also applied to participants with stroke: (1) residual hemiparesis; (2) the stroke occurred at least 4 days before the first assessment; and (3) functional restriction in the upper extremities.

**FIGURE 1:**
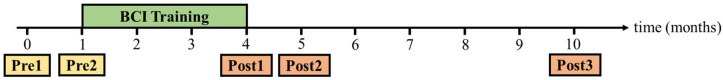
Timing of assessments (Pre-assessments 1 and 2 are yellow; Post-assessments 1, 2, and 3 are orange) and BCI Training period (green) for each patient.

Each participant received 3 months of BCI-supported MI training with 2 weekly sessions, 25 sessions in total. Two pre-assessments (Pre1 and Pre2) and three post-assessments (Post1, Post2, and Post3) were performed by two certified physiotherapists and were evaluated by the research team. Pre1 and Pre2 were scheduled 1 month and a few days before the intervention (respectively), while Post1, Post2, and Post3 were carried out a few days, 1 month, and 6 months after the intervention (respectively) (see Figure 1).

### Participants’ Baselines

Fifty-one stroke patients were enrolled on this study, 28 males and 23 females. The mean age was 60.52 years (*SD* = 16.65). The time since the stroke ranged from 3 to 377 months [median time 36.5 months, IQR = (21.00–79.00)]. The participants were classified in four groups based on their stroke diagnosis: Cortical, Subcortical, Cortical + Subcortical and Unknown. The most common type of stroke was Subcortical with 20 patients (39.2%), followed by Cortical + Subcortical with 12 patients (23.5%) and Cortical with 5 patients (9.8%). 14 patients where categorized as Unknown (27.5%).

Forty-five patients were in the chronic phase (88.2%), and six were in the subacute phase (11.8%). Thirty-four patients had a stroke in the right hemisphere (66.7%), and the stroke was in the left hemisphere in 17 patients (33.3%). Table 1 shows the participants’ baselines.

**TABLE 1:** Participants’ baselines.

Group	*n*	Age (*SD*) (years)	Time since stroke [months]	Male	Female	Affected side (Left–Right)
Cortical	5	54.00 (27.1)	59.4 (65.5)	4	1	3–2
Subcortical	20	63.05 (17.0)	59.7 (47.3)	10	10	13–7
Cortical + Subcortical	12	57.58 (14.6)	76.0 (83.4)	6	6	9–3
Unknown	14	61.79 (14.2)	61.5 (98.1)	8	6	9–5
All	51	60.52 (16.7)	64.04 (72.0)	28	23	34–17

### Functional and Behavioral Assessment

A series of functional and behavioral scales were administered in pre- and post-assessments. The primary outcome measure was the upper extremity section of the Fugl-Meyer assessment (FMA-UE, 66 max score). The score reflects impairment in upper limb functions, with lower scores corresponding to greater impairment, and is often used to assess the damage resulting from stroke and progress during therapy (Woytowicz et al., 2017). The grasp dexterity was assessed by the 9-Hole Peg Test (9-HPT) and Box and Block Test (BBT). 9HPT measures the time that the user needs to pick up nine small pegs from one box, place each peg in to one of nine holes into another box, and then return all pegs to the original box. The BBT measures the number of blocks that the patient can move from one container to another in 1 min while avoiding obstacles between the two containers (Mathiowetz et al., 1985). We used the Modified Ashworth Scale (MAS) to assess spasticity, in which low punctuations reflect less spasticity (Meseguer-Henarejos et al., 2018). The MASWrist was used to test wrist spasticity and the MASFingers scale tested finger spasticity. The Barthel Index (BI) is a questionnaire designed to test the patient’s ability to perform daily living activities (Quinn et al., 2011). The Fahn Tremor Rating Scale (FTRS) scores tremor intensity in the paretic limb (Fahn, 1993). The score ranges from 0 to 12 points, with a lower score indicating smaller tremor intensity. Sensitivity was measured with the Two Point Discrimination Test (TPDT), with a lower score indicating greater sensitivity (Wolny et al., 2017). We used the Stroop Color-Word Test (SCWT) and the Montreal Cognitive Assessment (MOCA) for cognitive assessment. The SCWT entails three different cards, each with a 10 × 10 matrix of words of color names, and the patient is asked to read as many words as possible in 45 s (Stroop, 1935). The first card is printed in black, the second card contains words printed in the same color (such as the word “BLUE” printed in blue), and the third card has words printed in a different color (such as the word “BLUE” printed in red). People with some types of cognitive dysfunctions will be able to read fewer words than healthy persons. The MOCA scale is widely used to assess the cognitive state of neurologic patients (Koski, 2013). This scale has 8 parts, and the total score ranges from 0 points to 30 points. Higher scores indicate better cognitive function, and a MOCA score above of 25 points is considered normal.

All patients answered a Self-Rated questionnaire (SRQ) with five parts: Pain (0–70 points); Function (0–70 points); Memory and thinking (0–70 points); Ability to be mobile at home and in the community (0–90 points); and Stroke recovery (0–10 points). Each of these parts has descriptions of different tasks. The patient estimates the difficulty in performing the task on a scale from 0–10, where 0 means “unable to do,” and 10 means “no difficulty.” The scale is different for the Pain part, where 0 means “none” and 10 means “extreme.”

### BCI System Description

**FIGURE 2:**
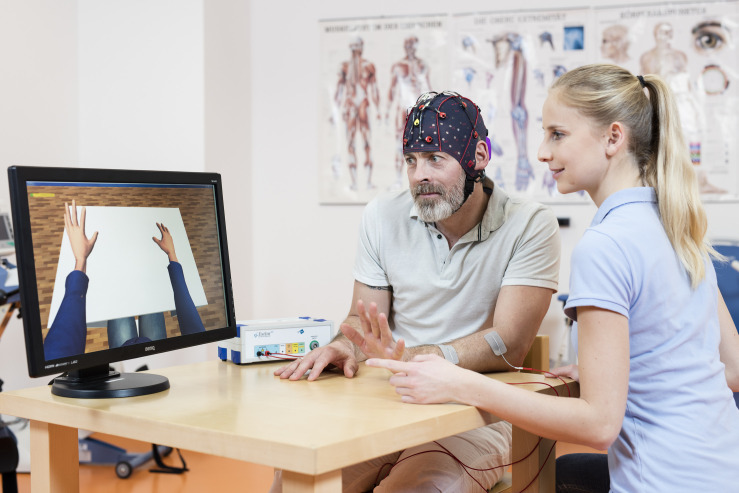
This photograph shows components of the BCI system used in this study, including a monitor with an avatar to instruct the patient and provide visual feedback. The EEG system measures the brain activity, which is analyzed by the BCI in real-time. As soon as the BCI system detects left- or right- hand movement imagination, the avatar moves the left or right hand and the left or right FES activates to produce hand movement. Copyright: g.tec medical engineering GmbH, republished with permission.

Patients were seated in a comfortable chair in front of an LCD screen with both arms resting on a desk. Patients wore EEG caps with 16 active electrodes (g.Nautilus PRO, g.tec medical engineering GmbH, Austria). EEG electrode positions were FC5, FC1, FCz, FC2, FC6, C5, C3, C1, Cz, C2, C4, C6, CP5, CP1, CP2, and CP6 according to the international 10/20 system. A reference electrode was placed on the right earlobe and a ground electrode at FPz (see Figure 2).

One pair of FES pads were placed on the skin over both the left and right wrist extensors. The two FES devices (g.Estim FES, g.tec medical engineering GmbH, Austria) were set to a frequency of 50 Hz and a rectangular pulse width of 300 μs. The stimulation amplitude (in mA) was adjusted to find the optimal movement produced by electrical stimulation in both the healthy and affected limbs without inducing pain or spasms.

All participants were instructed to imagine the dorsiflexion wrist movement according to the system indications. This is a type of mental imagery (MI) task. One session was composed by 240 MI repetitions on both hands, divided in 3 runs of 80 trials. Each session lasted about one hour, including time for preparation and cleaning.

**FIGURE 3:**
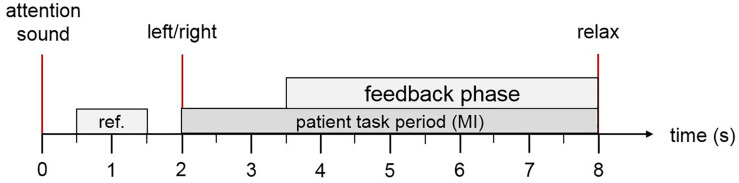
Trial description. The patient hears an attention sound at trial onset. At second 2, the system presents an arrow on the computer screen to instruct the patient to imagine left or right hand movement and a corresponding verbal instruction for left or right in the patient’s native language. During the feedback period, the FES and the virtual avatar are activated if the MI was classified correctly. At second 8, the patient hears a relax command.

The MI tasks were presented in pseudo random order with randomized inter-trial intervals. Figure 3 depicts the timing of each trial. Patients were first cued to the start of a trial with an attention beep. Two seconds later, an animated arrow in the avatar window pointed to the expected hand for MI. At the same time, an auditory instruction either left or right indicated the task of each trial. During the feedback phase, FES and avatar was triggered when the system detected MI of the correct hand. If no MI is detected, feedback is deactivated. Feedback was updated five times per second.

### Signal Processing

EEG signals were sent to a biosignal amplifier and were bandpass filtered (4th order Butterworth filter) between 8 and 30 Hz. Then, common spatial patterns (CSP) were applied to transform the data to a new matrix with minimal variance of one class and maximal variance of the other class (Blankertz et al., 2008). Each class reflects the MI of the cued hand vs. the MI of the other side. The CSP method calculated a 16 × 16 projection matrix from 16 EEG channels for each left and right trial *X*. This matrix is a set of spatial patterns that may reflect regional cortical activation during hand MI. The decomposition of a trial is written as *Z* = *WX*. This transformation projects the variance of *X* onto the rows of *Z* and results in 16 new time series. The columns of A = *W*^–^*^1^* are a set of CSPs and can be considered as time-invariant EEG distributions. The variance for left trials is largest in the first row of *Z* and decreases with the subsequent rows. The opposite occurs in a trial with right trials. The variances were extracted as reliable features of the newly calculated 16 time series for the binary classification (left vs. right).

According to Mueller-Gerking’s work, the optimal number of CSPs should be four (to reduce the dimensionality of EEG) (Muller-Gerking et al., 1999). Using an artifact corrected training set, *XT*, only the first and last two rows (*p* = 1, 2, 15, and 16) of *W* were used to process new input *X*. Then, the variance (*VARp*) of the times series was calculated for a time window *T*. After normalizing and log-transforming, four feature vectors were obtained via Eq. 1.

(1) $$f_{\text{p}}=\log\left(\frac{V\,A\,R_{p}}{\sum_{\text{p}=1}^{4}V\,A\,R_{p}}\right)$$

A linear discriminant analysis (LDA) classified each trial as either left or right MI. When the input signals were correctly classified according to the assigned task, the feedback devices were triggered. This online classification and control of the FES and avatar were updated every 25 ms.

We estimated offline classification accuracy via a 10-fold cross validation. This refers to partitioning a sample of movements into 10 complementary subsets and validating the analysis on one subset (called the validation set or testing pool) and training the CSPs and classifier on the other subsets (called the training pool).

The accuracy was calculated (in steps of half a second) for all trials in the testing pool within a 4.5 s time window beginning 1.5 s after the attention beep and ending with the end of the trial. For each step and each trial, the classification result is either 100 or 0%. The accuracy of all trials of the test pool is then averaged for each single step, resulting in accuracy levels ranging between 0 and 100%. After averaging all ten repetitions of the cross validation, the maximum value during the feedback phase was noted as the session accuracy.

### Statistical Analysis

The software used for the statistical analysis was MATLAB R2017b. We designated the mean of Pre1 and Pre2 as the baseline value for each outcome measure [PRE = (Pre1 + Pre2)/2]. Post-assessment was the outcome measure after completion of the 25 training sessions. The primary and secondary outcomes were statistically analyzed after a normal distribution was determined with the Shapiro-Wilk test. The significance threshold was set to α = 0.05. The statistical test was chosen according to the normality of the sample, the homogeneity of variance (Levene’s or Brown-Forsythe test of equal variance) and sample size. Descriptive statistics will be showed as mean and the standard deviation (SD), or the median with the inter-quartile rate (IQR) of 0.25 and 0.75.

A two-tailed paired sample *t*-test or a Wilcoxon signed rank test was used to investigate the outcome of changes between two different assessments in the same group of patients. We used the unpaired *t*-test or Mann-Whitney *U* test to compare the results of two different groups based on the mean accuracy obtained during the BCI training.

For multiple comparisons, p-values were corrected using the False Discovery Rate (FDR) described by Benjamini and Hochberg (1995), which explains that adjusted p-values can be greater than 1. All p-values greater than 1 were converted to 1.

First, we analyzed the functional improvement after the BCI therapy using paired comparison (*t*-test or Wilcoxon signed rank test) between PRE values and Post1 values. The second step was analyzing the long-term effects 1 month after therapy by comparing Post1 vs. Post2, and 6 months after the therapy by comparing Post1 vs. Post3.

We also studied the relation between MI accuracy and functional improvement. The accuracy threshold was defined at 80%. Based on this threshold, each patient was classified as either above or below threshold using the median of the MI accuracy during the therapy.

Finally, we studied the relationship between functional improvement and the degree of impairment. Woytowicz et al. (2017) categorized the degree of impairment based on the FMA-UE score. Following the same criteria, we classified our participants in three groups based on the baseline FMA-UE score: severely impaired (0–28 points), moderately impaired (from 29 to 42 points) and mildly impaired (from 43 to 66 points).

## Results

### Functional Improvement After BCI Therapy

The results in this section summarize differences from the PRE to Post1 assessments across different tests. Motor function was mainly assessed by FMA-UE, BBT, and 9HPT, all of which showed some significant improvement after the therapy. Table 2 shows the results.

**TABLE 2:** Summary of the functional improvement after BCI treatment.

Scale		*n*	PRE	Post	Δ		*P*	
			Median [IQR]	Median [IQR]	Median [IQR]	Mean (*SD*)	No adj	Adj
BI		51	90 [70–95]	95 [67.5–100]	0 [0–5]	2.62 (*SD* = 5.82)	0.002	0.083
FTRS	Healthy	50	0 [0–0.5]	0 [0–0]	0 [0–0]	−0.25 (*SD* = 0.66)	0.008	0.163
	Paretic	50	12 [6–12]	11 [4–12]	0 [−1.5–0]	−1 (*SD* = 2.42)	0.003	0.090
MAS	Wrist	51	2.5 [0.63–3.5]	1 [0–3]	−0.5 [−1.44–0]	−0.72 (*SD* = 0.83)	< 0.001	<0.001
	Fingers	51	2.5 [1–3.5]	2 [1–3]	−0.5 [−1–0]	−0.63 (*SD* = 0.82)	< 0.001	<0.001
BBT	Healthy	42	51.25 [43–64]	59 [48–72]	6.25 [1.5–9]	6.29 (*SD* = 7.25)	< 0.001	<0.001
	Paretic	43	0 [0–6]	0 [0–5.75]	0 [0–1.5]	1.5 (*SD* = 3.13)	0.006	0.129
9HPT	Healthy	49	23 [19.79–28.5]	22 [18.75–25]	−1.55 [−3.5—0.43]	−2.05 (*SD* = 3.5)	< 0.001	0.010
	Paretic	9	190 [154.13–364.59]	170.32 [110.25–195.5]	−52 [−172.01—26.5]	−75.58 (*SD* = 118.18)	0.091	1.000
TPDT	H. Thumb	41	3.5 [2.88–4]	3 [2–4]	−0.5 [−1–0]	−0.59 (*SD* = 1.34)	0.003	0.090
	H. Index	42	3.5 [3–4]	3 [2–3]	−0.5 [−1–0]	−0.43 (*SD* = 0.77)	0.001	0.057
	P. Thumb	24	4.5 [4–5.5]	3 [2–4]	−1 [−2—0.25]	−1.4 (*SD* = 2.16)	0.003	0.090
	P. Index	26	3.75 [3–5]	3 [3–4]	0 [−1–1]	−0.31 (*SD* = 1.66)	0.388	1.000
FMA-UE		51	19 [9.63–33.88]	22 [12–41.75]	4 [1–8]	4.68 (*SD* = 4.92)	< 0.001	<0.001
FMA-LE		19	20 [12.63–24.38]	20 [13–25.5]	0 [−0.38–1]	0.45 (*SD* = 3.48)	0.582	1.000
SRQ	Pain	41	30.5 [19.75–37.13]	23 [17.75–39.25]	0 [−7.25–3.5]	−1.2 (*SD* = 11.96)	0.528	1.000
	Function	41	4 [0–13.88]	6 [0–20.25]	0 [−0.38–4.13]	1.57 (*SD* = 14.65)	0.449	1.000
	Memory	40	60.5 [45.75–70]	65 [43.5–70]	0 [−3–5.25]	1.03 (*SD* = 17.72)	0.581	1.000
	Mobility	41	71 [38.38–80]	77 [44.5–84]	1 [−1.38–7]	4.94 (*SD* = 20.94)	0.051	0.768
	Recovery	39	5 [4.13–6.38]	7 [5–7]	1 [0–2]	0.87 (*SD* = 1.62)	0.003	0.090
MOCA		10	27 [21.5–28]	27.5 [26–29]	2 [1–5]	2.15 (*SD* = 3.33)	0.109	1.000
SCWT	Word	10	73.75 [54–100.5]	75.5 [64–108]	4.25 [−2–10]	3.95 (*SD* = 6.95)	0.106	1.000
	Color	10	73 [59–94]	71 [62–101]	3.75 [1–8]	4 (*SD* = 5.67)	0.053	0.768
	Color-Word	10	22.25 [10.5–34.5]	31.5 [18–34]	6.75 [2–8]	5.75 (*SD* = 4.28)	0.002	0.083

*BI, Barthel Index; FTRS, Fahn Tremor Rating Scale; MAS, Modified Ashworth Scale; BBT, Box and Block Test; 9HPT, Nine Hole Peg Test; TPDT, Two Point Discrimination Test; FMA-UE, Fugl Meyer Assessment Upper Extremity; FMA-LE, Fugl Meyer Assessment Lower Extremity; SRQ, Self Rated Questionnaire; MoCA, Montreal Cognitive Assessment; SCWT, Stroop Color Word Test; The abbreviations “H.” and “P.” in the TPDT test row mean “Healthy” and “Paretic” respectively. The last column shows the P-values non-adjusted, “No adjs,” and adjusted, “Adj” by the FDR method.*

**FIGURE 4:**
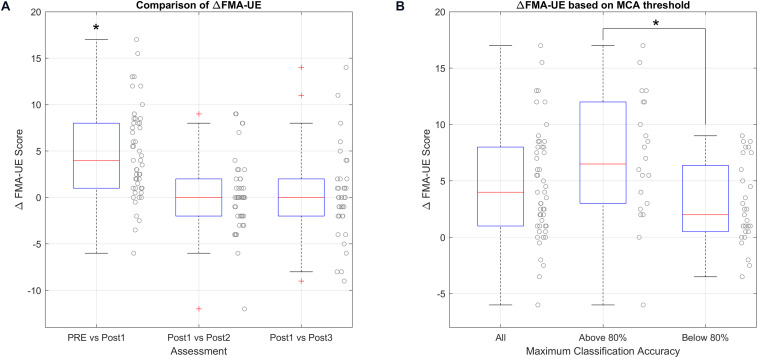
Analysis of ΔFMA-UE. (*) marks significant changes. **(A)** Shows the FMA improvement before and after the therapy, and the functional changes at different times after the last therapy session. **(B)** Shows the relationship between the improvement in the FMA-UE scale and the accuracy threshold.

The functional baseline in the upper extremity assessed by the FMA-UE was 19 points [9.63–33.88] on a scale with a maximum score of 66 points. The Wilcoxon signed rank test shows that there is a significant improvement in FMA-UE after the therapy [ΔFMA-UE = 4 (1–8), *P* < 0.001]. The mean improvement is 4.68 (*SD* = 4.92). Figure 4A shows the comparison of the FMA-UE before and after the therapy. In the first box of this figure (PRE vs. Post1), we can see that the points cloud is above 0 points, which means that in general all patients improved in this scale and only 5 patients decreased after the therapy. The FMA-UE score after the therapy was 22 points [12–41.75].

The functionality in the lower extremity before the therapy, assessed by FMA-LE in 19 patients, was 20 points [12.63–24.38]. Changes after therapy were not significant ΔFMA-LE = 0 [−0.38 to 1], *P* = 1.000.

The values of the BBT in the healthy hand before the therapy was 51.25 blocks [43–64]. This scale showed a significant change after the therapy, ΔBBT-healthy = 6.25 [1.5–9], *P* < 0.001, and the mean delta was 6.29 points (*SD* = 7.25). The basal values of the BBT of the paretic hand was 0 blocks [0–6], and this scale did not show significant changes in the BBT after the therapy, ΔBBT-paretic = 0 [0–1.5], *P* = 0.129.

Patients needed 23.00 s on average [19.79–28.5] to perform the 9HPT with the healthy hand before the therapy. After the therapy, there was a significant reduction of the time needed to perform the test, Δ9HPT-healthy = −1.55 [−3.5 to −0.43], *P* = 0.010, and the mean change was −2.05 s (*SD* = 3.5). In the paretic hand, only 9 patients could perform the 9HPT test at the beginning, and the mean needed time to do the test was 190 s [154.13–364.59]. There were no significant changes after the therapy, Δ9HPT-paretic = −52.00 [−172.01 to 26.5], *P* = 1.000.

Before the therapy, the patients reported some degrees of spasticity with 2.5 points [0.63–3.5] in the wrist and 2.5 points [1– 3.5] in the fingers. After the therapy, the MAS scale showed a significant reduction of the spasticity in the fingers, ΔMAS-fingers = −0.5 [−1 to 0], *P* < 0.001, and the mean delta was −0.63 (*SD* = 0.82), and the wrist, ΔMAS-wrist = −0.5 [−1.44 to 0], *P* < 0.001, and the mean change was −0.72 (*SD* = 0.83).

The degree of tremor did not show a significant change using the FTRS. The tremor in the healthy hand before the therapy was very low, 0 points [0–0.5], and consequently the change after the therapy was not significant, ΔFTRS-Healthy = 0 [0–0], *P* = 0.163. In the paretic hand, the tremor before the therapy was very high, 12 points [6–12], and the change after the therapy was not significant, ΔFTRS-Paretic = 0 [−1.5 to 0], *P* = 0.090.

The sensory acuity in the healthy hand before the therapy was 3.5 mm [2.88–4] in the thumb, and 3.5 mm [3–4] in the index. The changes after therapy were not significant in the healthy hand, ΔTPDT-healthy-thumb = −0.5 [−1 to 0], *P* = 0.090, ΔTPDT-healthy-index = −0.5 [−1 to 0], *P* = 0.057. The sensitivity in the paretic thumb before therapy was 4.5 mm [4–5.5], and was 3.75 mm [3–5] in the index of the paretic hand. The changes on this scale were also not significant for the paretic hand, ΔTPDT-paretic-thumb = −1 mm [−2 to −0.25], *P* = 0.090, ΔTPDT-paretic-index = 0 mm [−1 to 1], *P* = 1.000.

The baseline values of the BI were 90 points [70–95] up to 100. The BI showed no significant changes after the therapy, ΔBI = 0 [0–5], *P* = 0.083.

The baseline cognitive assessment using MOCA was 27 points [21.5–28], and the values after the therapy did not change significantly ΔMOCA = 2 points [1–5], *P* = 1.000. The other cognitive scale, SCWT, had the following baseline values: 73.75 words [54–100.5] for the Word card; 73 words [59–94] for the Color card; and 22.25 words [10.5–34.5] for the Color-Word card. No significant changes were detected on this test, ΔWord = 4.25 words [−2 to 10], *P* = 1.000, ΔColor = 3.75 words [1–8] *P* = 0.768, and ΔColor_Word = 6.75 words [2–8], *P* = 0.083.

Finally, the baseline values of the SRQ were: Pain = 30.5 points [19.75–37.13], Function = 4 points [0–13.88], Memory = 60.5 points [45.75–70], Mobility = 71 points [38.38–80], Recovery = 5 points [4.13–6.38]. No significant changes were detected on this questionnaire after the therapy; ΔPain = 0 points [−7.28 to 3.5] with *P* = 1.000, ΔFunction = 0 points [−0.38 to 4.13] with *P* = 1.000, ΔMemory = 0 points [−3–5.25] with *P* = 1.000, ΔMobility = 1 point [−1.38 to 7] with *P* = 0.768 and ΔRecovery = 1 point [0–2] with *P* = 0.090.

### Functional Outcomes in the Long Term

The analysis of the long-term effects based on comparisons between the Post2 and Post3 assessments did not show significant results. Table 3 shows the results.

**TABLE 3:** Analysis of long-term effects.

Scale		*n*	ΔPost1-Post2	*P*	*n*	ΔPost1-Post3	*P*
			Median [IQR]	Adj		Median [IQR]	Adj
BI		46	0 [0–0]	1.000	38	0 [0–0]	1.000
FTRS	Healthy	45	0 [0–0]	1.000	37	0 [0–0]	1.000
	Paretic	45	0 [0–0]	1.000	37	0 [−1–1]	1.000
MAS	Wrist	46	0 [0–0]	1.000	38	0 [0–0]	1.000
	Fingers	46	0 [0–0]	1.000	38	0 [−0.5–0]	0.580
BBT	Healthy	44	2 [−3–4.5]	1.000	37	0 [−3–4]	1.000
	Paretic	44	0 [0–0]	1.000	37	0 [0–0.25]	1.000
9HPT	Healthy	45	−1 [−2–0.52]	0.119	37	0 [−2.25–1.21]	1.000
	Paretic	9	−28 [−61.67–39.25]	1.000	8	3.89 [−29.5–64.15]	1.000
TPDT	H. Thumb	41	0 [−0.25–0.25]	1.000	36	0 [0–1]	0.838
	H. Index	42	0 [0–1]	1.000	35	0 [−0.75—-1]	1.000
	P. Thumb	23	0 [−1–0]	1.000	17	0 [−0.25–2]	1.000
	P. Index	21	0 [−0.25–0]	1.000	20	1 [0–2]	0.838
FMA-UE		46	0 [−2–2]	1.000	38	1 [−3–2]	1.000
FMA-LE		19	0 [0–0]	1.000	14	1.5 [0–3]	0.319
SRQ	Pain	41	0 [−4–3]	1.000	35	0 [−4.75–1.75]	1.000
	Function	41	0 [0–2.25]	1.000	35	0 [−2–1.75]	1.000
	Memory	41	0 [−0.25–3]	1.000	35	0 [0–10]	0.768
	Mobility	41	0 [0–3.75]	0.768	35	2 [0–6]	0.064
	Recovery	41	0 [−1–0]	1.000	35	0 [−1–0]	1.000
MOCA		13	0 [−1–1]	1.000	12	1 [0–2]	0.163
SCWT	Word	13	2 [−0.25–9.5]	1.000	12	2.5 [−1.5–9]	1.000
	Color	13	−4 [−11.5–5.25]	1.000	12	7.5 [−4.5–15.5]	1.000
	Color-Word	13	0 [−2.5–3.75]	1.000	12	3.5 [−1–6.5]	0.768

The primary measure, FMA-UE, did not show significant changes in Post2. The median change between Post1 and Post2 was 0 points [−2 to −-2], *P* = 1.000. The changes 6 months after the therapy are also not significant, 1 point [−3 to 2], *P* = 1.000. Figure 4A shows these results.

### Functional Improvement and MI Accuracy

Patients with median MI accuracy above of 80% increased their FMA-UE score by 6.5 points [3–12], and the mean was 6.97 points (*SD* = 5.51). For patients that were below 80% median MI accuracy, the median improvement in FMA-UE was 2 points [0.5–6.38], and the mean was 2.93 points (*SD* = 3.62).

The unpaired *t*-test for equal variances shows that patients with median MI accuracy above 80% improved 3.16 points more [95% CI; (1.47–6.62), *P* = 0.003] than the other patients. Figure 4B shows the result of the comparison.

### Functional Improvement and Degree of Impairment

**FIGURE 5:**
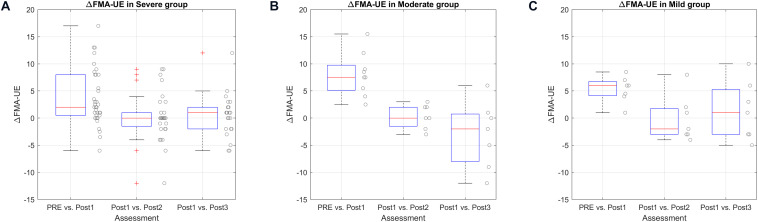
ΔUE-FM score according to impairment level represented by boxplots (box: 25–75 percentile, whiskers: 5–95 percentiles). Single data are shown, scattered along the y-axis for a better visualization. **(A)** Comparison of delta FMA-UE score in severe group. **(B)** Comparison of delta FMA-UE score in moderate group. **(C)** Comparison of delta FMA-UE score in mild group.

First, we present the improvement within groups to assess whether all groups improved significantly after the BCI therapy. Figure 5 shows these comparisons.

In the Severe group (35 patients), the PRE vs. Post1 median change was 2 points [0.5–8], and the mean change was 3.70 points (*SD* = 5.18), *P* = 0.006. The delta between Post1 and Post2 was 0 points [−1.5 to 1], *P* = 1.000, and the delta between Post1 and Post3 was 1 point [−2 to 2], *P* = 1.000.

The Moderate group (9 patients) showed a significant improvement between PRE and Post1 of 7.5 points [5.13–9.75], *P* = 0.033, and the mean was 8.00 points (*SD* = 3.98). The change between Post1 and Post2 was 0 points [−1.5 to 2], *P* = 1.000, and the delta between Post1 and Post3 was −2 points [−8 to 0.75], *P* = 1.000.

Finally, the Mild group (7 patients) also showed significant differences between PRE and Post1. The median difference was 6 points [4.13–6.75], *P* = 0.015, and the mean improvement was 5.29 points (*SD* = 2.41). The median change between Post1 and Post2 was −2 points [−3 to 1.75], *P* = 1.000, and the delta between Post1 and Post3 was 1 point [−3 to 5.25], *P* = 1.000.

The comparison between groups using a One-Way ANOVA with the data PRE vs. Post1 showed non-significant differences between groups, *F* = 3.025 and *P* = 0.058.

### General Improvement Before vs. After the Therapy

**FIGURE 6:**
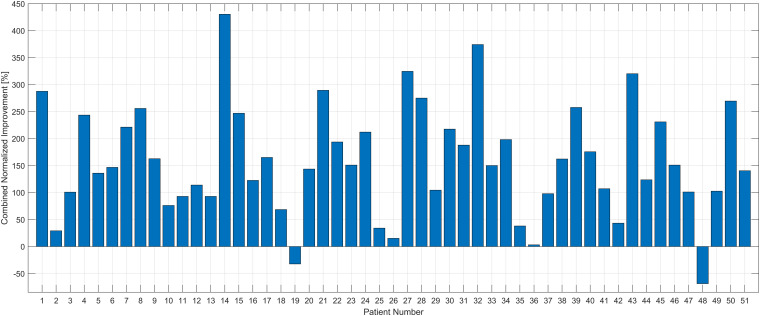
Combined normalized improvement [%] of each patient. The improvement (differences between Pre and Post1) of each scale was normalized and summed up for the therapy effect in total. All the patients had positive outcomes in this total scale except patients #19 and #48.

Figure 6 shows the combined change of each patient including all the scales. The normalization shows that almost all patients had an increase of the functional skills, and only patients 19 and 48 showed a negative outcome in some scales.

## Discussion

The objective of this study was to analyze the effects of the MI BCI therapy for upper extremity motor rehabilitation in stroke survivors. Fifty-one stroke patients with hemiparesis in the upper extremity completed the study and were categorized according to their stroke location, level of impairment determined by the baseline FMA-UE, and time since stroke.

The primary measure, FMA-UE, assessed upper limb functionality and showed that patients improved upper extremity motor function significantly by 4.68 points (*SD* = 4.92). This mean change is above the Clinically Important Difference (CID) (Page et al., 2012). These BCI therapy effects persisted throughout the post-project assessments, with no significant changes after 1 month nor after 6 months. The majority of the patients (84.3%) improved at least 1 point in the FMA score. Among patients who improved, the mean improvement was 5.88 points (*SD* = 4.30). 5 patients decreased at least 1 point in FMA score, and the mean decrease in this group was −2.9 points (*SD* = 2.04). The FMA score did not change in the remaining 3 patients.

This motor improvement was preceded by the reduction of the spasticity in the fingers and the wrist. Usually, the patients verbally reported a reduction of the spasticity in the affected hand during the therapy. This fact seems be related with performance in the other motor tests like BBT and 9HPT. Another important finding is that the spasticity did not increase some months after therapy ended. This will be discussed later.

The spasticity reduction and the significant improvement in the upper extremity function may explain the improvement in grasp ability. Thirty five patients (68.6%) improved their BBT scores with the healthy hand, and 13 (25.5%) improved with the paretic hand. Two patients could not perform the BBT before the therapy due to the severity of the motor impairment, but both patients could move at least one block after the therapy. In the 9HPT, 38 out of 49 patients (77.5%) improved coordination and speed in the healthy hand. Performing this test with the paretic hand was challenging for most patients. Only 9 patients could perform the 9HPT before the therapy, and 8 of them reduced the needed time to complete the 9HPT. One patient was unable to perform the 9HPT with the paretic hand prior to therapy, but after the therapy, this patient could complete this test in 324 s.

As mentioned above, these results show some significant improvements in the performance of the healthy hand. It is well known that coordinated movements require interactions between two hemispheres and primary motor areas. The reduction of the time in the 9HPT of the healthy hand, and the improvement in the BBT, could result from improved inter-hemisphere interactions. Other studies suggest that bimanual coordination between paretic and non-paretic hands is highly associated with motor recovery (Swinnen and Wenderoth, 2004; Lai et al., 2019).

Nevertheless, the motor system improvements were not evident in other scales. The FMA improvements should correspond to increases in scores for daily living activities in Post1, Post2, and Post3. This expected functional improvement was not apparent in the Post1 or in the long-term analysis of BI, probably because the roof effect of this scale. The BI score decreased in 3 patients, while 23 patients reported positive changes in the BI after the therapy and 25 patients did not show changes in this parameter. Similarly, motor changes are usually accompanied by changes in the sensory system, but sensitivity did not change significantly. After alpha correction, the TPDT changes are not significant. All these inconsistent results (no improvement in daily living activities or sensitivity) might be explained with our selection of scales in this study. Maybe the measure of the daily living activities or the sensitivity is not accurate enough. This could explain who only 26 patients could perform the TPDT in the paretic hand.

The feedback that patients received was related to the maximum MI accuracy. Patients that were more focused during the therapy might be able to attain better MI accuracy and use the BCI system more effectively. We analyzed the relation between BCI performance and the functional improvement assessed by the primary measure, FMA-UE. Figure 4B shows that patients with high MI accuracy (above 80%) showed greater improvement. Thus, MI accuracy may be a useful tool to help predict outcomes and help both patients and therapists identify and address non-compliance.

One of the patient’s main tasks is learn how perform MI most effectively, and the role of the healthy hand may be important. Patients might relearn MI by imagining or performing the movement with the healthy side and applying the same mental strategies in the paretic side. This approach may be most effective when the feedback is equally applied to both sides.

We also analyzed the functional improvement based on stroke severity, shown in Figure 5. The functional improvements in all groups were significant after the therapy. Hence, BCI therapy could be helpful independent of the previous functional impairment. The comparison between groups to determine which group can benefit most from the BCI therapy was not significant. Our results with the methods used here provide no evidence that one group improved more than any other.

Functional improvement was not correlated with stroke stage either. Patients in the chronic stage could improve motor function in the paretic side, which is not commonly accepted. These results support other work indicating that a BCI can play an important role in cortical reorganization that underlies functional improvement (Dobkin, 2007; Biasiucci et al., 2018; Cervera et al., 2018). The improvements experienced by the BCI-treatment are due to neuroplastic changes in the central nervous system caused by closed-loop learning (Wolpaw, 2007; Biasiucci et al., 2018; Cervera et al., 2018), rather than improvements due to the effects of muscle electrostimulation that disappear over time. The long-lasting effects of the FES treatment alone are under discussion (Howlett et al., 2015; Eraifej et al., 2017), but the real-time synchronization of MI, immersive avatar therapy and electrical stimulation could be a powerful combination to bolster Hebbian plasticity underlying recovery.

Some limitations and opportunities for future research should be mentioned. First, this study did not employ a control group, which limits direct comparisons between the approach used here and conventional rehabilitation approaches. Randomized clinical trials that consider different treatment arms and compare BCI versus non-BCI therapies should further elucidate the best approaches to rehabilitation. Prior studies have already shown that BCI therapy can yield greater functional improvement than conventional therapy. The objective of this investigation was instead to explore the effects of the approach used here to assess whether results are similar to other studies and may have clinical value. Second, not all patients could perform all the tests both before and after the therapy. This limitation led to statistical analyses with a low sample size because we disregarded those subjects who could not do the test. Third, this study used the term “long-term,” but we have not yet explored effects beyond 6 months after therapy ends. Further research could explore whether the approach used here, and related approaches, yield benefits that persist even longer. Fourth, while we used a broad range of scales, different scales and assessment methods could yield different results. Fifth, emerging qEEG approaches (Sebastián-Romagosa et al., 2020) or other analysis tools could also provide further insight.

## Data Availability Statement

The datasets generated for this article are not readily available because patients’ data need to be treated according to current data protection laws and ethical guidelines. Requests to access the datasets should be directed to MS-R, sebastian@gtec.at and CG, guger@gtec.at.

## Ethics Statement

The studies involving human participants were reviewed and approved by the Ethikkommission des Landes Oberösterreich, Austria. The patients/participants provided their written informed consent to participate in this study. Written informed consent was obtained from the individual(s) for the publication of any potentially identifiable images or data included in this article.

## Author Contributions

MS-R and WC participated in the data acquisition, performed the analysis, and did the main contribution to the manuscript writing. RO supervised the whole process, data acquisition, analysis, and manuscript revision. NM participated to the data acquisition. TV and KK participated in the manuscript revision, supervised the whole process, and provided clinical input. BZA provided the scientific input and contributed to the manuscript writing. CG supervised the whole project and reviewed the manuscript. All authors contributed to the article and approved the submitted version.

## Conflict of Interest

MS-R, WC, RO, and NM were employed at g.tec medical engineering. CG was CEO of g.tec medical engineering, who developed and sells the BCI system used on this study. The remaining authors declare that the research was conducted in the absence of any commercial or financial relationships that could be construed as a potential conflict of interest. The reviewer YZ declared a past co-authorship with one of the authors BZA to the handling editor.
